# Controllable Synthesis and Charge Density Wave Phase Transitions of Two-Dimensional 1T-TaS_2_ Crystals

**DOI:** 10.3390/nano13111806

**Published:** 2023-06-05

**Authors:** Xiaoguang Pan, Tianwen Yang, Hangxin Bai, Jiangbo Peng, Lujie Li, Fangli Jing, Hailong Qiu, Hongjun Liu, Zhanggui Hu

**Affiliations:** Tianjin Key Laboratory of Functional Crystal Materials, Institute of Functional Crystals, School of Materials Science and Engineering, Tianjin University of Technology, Tianjin 300384, China

**Keywords:** 1T-TaS_2_, CVD, resistance measurements, Raman spectra, NC/CCDW phase transition

## Abstract

1T-TaS_2_ has attracted much attention recently due to its abundant charge density wave phases. In this work, high-quality two-dimensional 1T-TaS_2_ crystals were successfully synthesized by a chemical vapor deposition method with controllable layer numbers, confirmed by the structural characterization. Based on the as-grown samples, their thickness-dependency nearly commensurate charge density wave/commensurate charge density wave phase transitions was revealed by the combination of the temperature-dependent resistance measurements and Raman spectra. The phase transition temperature increased with increasing thickness, but no apparent phase transition was found on the 2~3 nm thick crystals from temperature-dependent Raman spectra. The transition hysteresis loops due to temperature-dependent resistance changes of 1T-TaS_2_ can be used for memory devices and oscillators, making 1T-TaS_2_ a promising material for various electronic applications.

## 1. Introduction

Two-dimensional (2D) layered materials have exhibited novel physical properties different from bulk materials due to their atomically low thickness and high carrier mobility. Among them, low-dimensional strongly correlated electron systems, such as 1T-TaS_2_, 2H-TaSe_2_, 2H-NbSe_2_, and 1T-TiSe_2_, have unique electronic structures and rich extraordinary physical properties, including superconductivity [1], charge density wave order [2,3], ferromagnetism [4], and catalytic activity [5]. As a charge density wave (CDW) material, 1T-TaS_2_ has attracted much attention during the past years owing to its abundant phases, such as 1T, 2H, and 3R, with various stacking [6,7,8,9,10]. It has extensive prospects for applications as electronic, magnetic, and energy conversion devices, such as high-performance oscillators [6], fast memories [7], solar cells [8], humidity sensors [9,11,12,13], and high-efficiency electrocatalysts [10,14,15]. Recently, the ferroic character of 2D 1T-TaS_2_ was established by revealing the hysteretic electrical switching of the ferro-rotational order through the observation of its domains and domain wall propagation [16].

Unlike the Peierls instability mechanism, electron–phonon interaction plays a crucial role in driving CDW instabilities [17,18]. The instability of CDW featured by periodic lattice distortion (PLD) was found to be dependent on the temperature, which affected spatial modulation of carrier density at low temperatures and produced a metastable phase [19,20,21]. Below 180 K, a commensurate CDW (CCDW) phase was revealed [22], in which a $\sqrt{13}$ × $\sqrt{13}$ superlattice was formed when 12 Ta atoms on the outside shrunk to the central 13th Ta atom to form a “Star of David” (SOD) structure [23]. After the CCDW phase transformation, the bandgap opening made 1T-TaS_2_ a Mott insulator [24]. At 180 K, a nearly commensurate CDW (NCCDW) phase was formed with partial structures being commensurate to the original lattice, resulting in the metallic CCDW phase by reducing insulating domain walls. When further increasing to near 350 K, 1T-TaS_2_ distorted into an incommensurate CDW (ICCDW) phase, in which atoms were shifted from their original lattice positions. In addition to thermal excitation, CDW phase transition can be induced in other ways, including photoinduced phase transition [25] and electron-induced phase transition [26,27]. Electrical measurement shows the sudden change in resistance and the hysteresis window [28].

In recent years, chemical vapor deposition (CVD) has been used as a mature strategy for controllable synthesis of high-quality 1T-TaS_2_. The preparation of 2D TaS_2_ crystals with different substrates has been reported, such as Au [29,30,31], SiO_2_/Si [32], hexagonal boron nitride [33], sapphire [34], etc. However, the interaction between substrate and sample due to the charge transfer [35] inevitably affects CDW performance. Among these substrates, mica has excellent epitaxial growth characteristics and lattice adaptation degree. Here, we report the thickness-controllable growth of 1T-TaS_2_ on mica substrates by ambient pressure CVD (APCVD) and studies on CDW phase transitions. The structure of 1T-TaS_2_ was confirmed by Raman, X-ray diffraction (XRD), X-ray photoelectron spectroscopy (XPS), and high-resolution transmission electron microscope (HRTEM) measurements. After structural characterization, temperature-dependent resistance and temperature-varying Raman measurements were used to characterize the CDW phase transition under thermal excitation. 

## 2. Materials and Methods

### 2.1. Characterizations of As-Grown 2D 1T-TaS_2_ Crystals 

Morphologies and thicknesses of 2D 1T-TaS_2_ crystals were checked with an atomic force microscope (AFM, Bruker Corp., Billerica, MA, USA, Dimension Icon). The micro-Raman tests were performed with a confocal microscope-based Raman spectrometer (ALPHA 300, WITec Corp., Ulm, Germany) under an excitation laser at 532 nm. The temperature-dependent Raman spectra were collected in a custom-made vacuum thermostat ranging from 80 K to 260 K. The binding energies of elements were obtained by XPS measurements on as-grown samples (ESCALAB 250 Xi, Thermo Scientific Corp., Waltham, MA, USA). Before the TEM (Talos F200 X, FEI Corp., Hillsborough, OR, USA) measurements, the as-grown 1T-TaS_2_ crystals were transferred onto micro-grid-supported Cu grids via a typical polymethyl methacrylate (PMMA)-assisted transfer method [36]. The as-grown samples were spin-coated with PMMA (950 K, A4, Allresist Corp., Straussberg, Berlin, Germany) at 6000 rpm for 60 s, followed by drying at 180 °C for 10 min. Then, the samples supported by PMMA film were lifted up with tweezers under deionized water, and then they were collected by micro-grid-supported Cu grids. Finally, the PMMA film was removed via dissolution with acetone for about 10 min and dried by flowing Ar gas. HRTEM images and selected area electron diffraction (SAED) patterns were collected by a Talos F200X transmission electron microscope operated at 200 kV. 

### 2.2. Device Fabrication of 1T-TaS_2_ Electrical Devices

For individual TaS_2_ flakes, the electrodes were patterned by electron beam lithography (EBL). Five nanometers Ti and 50 nm Au were electron-beam evaporated for contacts. The devices were put in a vacuum (Janis ST500 probe station, <10^−5^ Torr) and measured by an Agilent B1500A semiconductor device analyzer. Low-temperature resistance measurements were performed in a physical property measurement system (PPMS, Quantum Design, Inc., San Diego, CA, USA) under liquid He-purged conditions.

## 3. Results and Discussion

**Figure 1:**
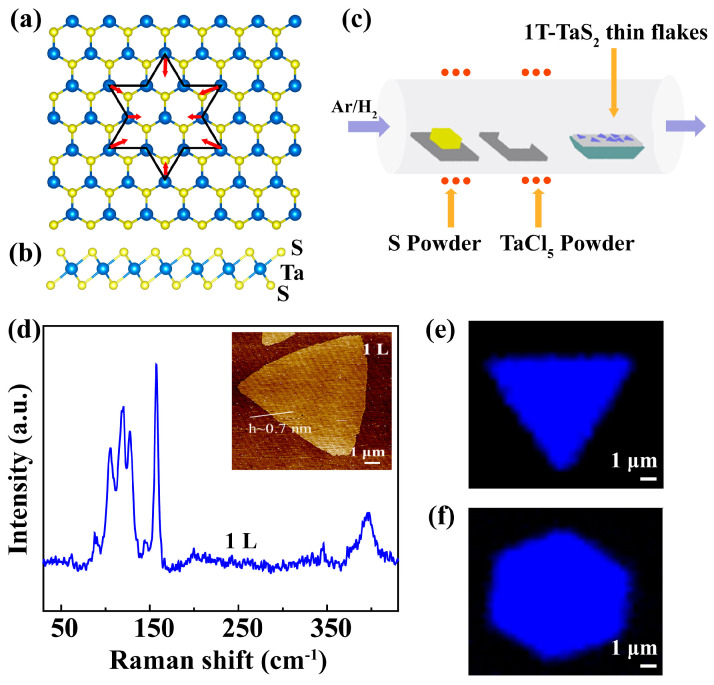
Synthesis of 1T-TaS_2_ crystal via APCVD method. Top−view (**a**) and side view (**b**) of the atomic structures of a 1T-TaS_2_ crystal. (**c**) Schematics for the APCVD growth of 1T-TaS_2_ on a mica substrate. (**d**) A Raman spectrum collected at room temperature. An AFM image in the inset demonstrates the height of 0.7 nm for the monolayer, with a scale bar of 1 μm. (**e**,**f**) Raman maps of 1T-TaS_2_ with different shapes.

The top-view and side-view atomic models of 1T-TaS_2_ are shown in Figure 1a,b. Ta atoms with the central octahedral arrangement are sandwiched between two S atom layers, demonstrating an ABC-type stacking [37]. The electron–phonon coupling-induced SOD structure at a low temperature is presented in Figure 1a, with red arrows indicating the shrinking direction of Ta atoms. At a temperature below 180 K, the 1T-TaS_2_ crystal is filled with SOD cluster, while at room temperature, the 1T-TaS_2_ crystal can transform from the CCDW to the NCCDW phase, which is only partially filled by satellite domains [38].

Two-dimensional 1T-TaS_2_ crystals were grown by the APCVD method, as schematically shown in Figure 1c. The mica was employed as a substrate placed in the deposition area. Tantalum pentachloride and sulfur powders were employed as the sources placed upstream outside the furnace, which were independently heated by two different heaters. Then, under the H_2_/Ar (5%H_2_) mixed carrier gas, the furnace was heated to 1100 K for growth. During the growth, the Ta flux was controlled while the S flux was kept continuous. By controlling the supply time for Ta and heating time, high-quality 1T-TaS_2_ crystals with various layer thicknesses were grown. The Raman spectrum of 1T-TaS_2_ monolayers collected at room temperature is shown in Figure 1d, in which peak wavenumbers less than 150 cm^−1^ were related to the tantalum atoms while phonon modes within 220−320 cm^−1^ were more associated with sulfur atoms [38]. The inset presents an AFM image of an as-grown 1T-TaS_2_ crystal with a lateral size of about 10 μm on which the Raman spectrum was collected. The main Raman phonon modes of 1T-TaS_2_ include A_1g_ modes of 71, 78, and 117 cm^−1^ and E_g_ modes of 60 and 90 cm^−1^. Figure 1e,f shows Raman maps of 1T-TaS_2_ crystals with unicolor trigonal and hexagonal shapes, indicating excellent crystallinity of the as-grown crystals.

**Figure 2:**
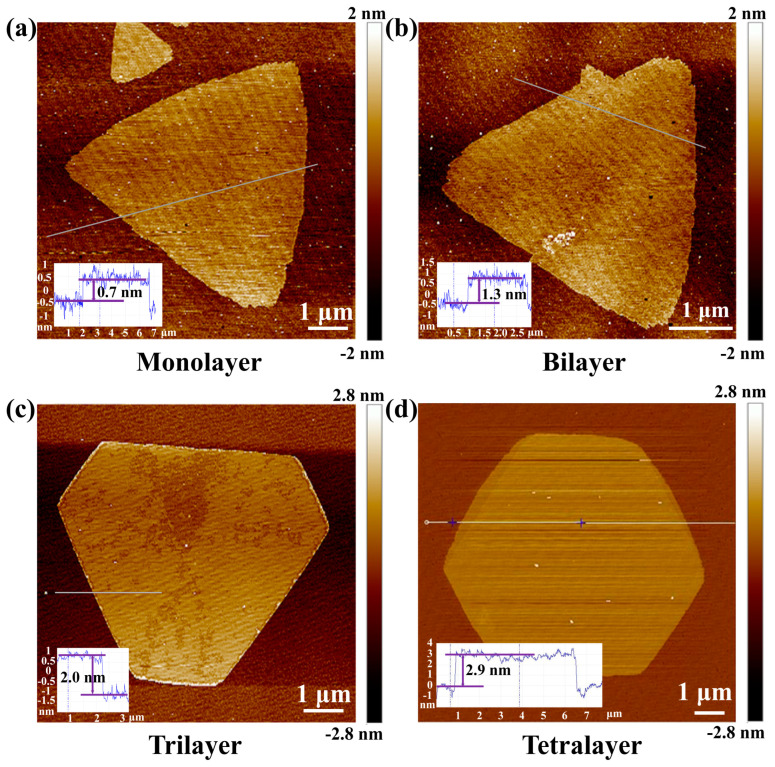
Layer−controlled synthesis of 1T-TaS_2_ crystal via APCVD method. AFM images for the monolayer (**a**), bilayer (**b**), trilayer (**c**), and tetralayer (**d**) are presented, and the line profiles are shown in the corresponding inset, respectively. The scale bar is 1 μm in each image.

**Figure 3:**
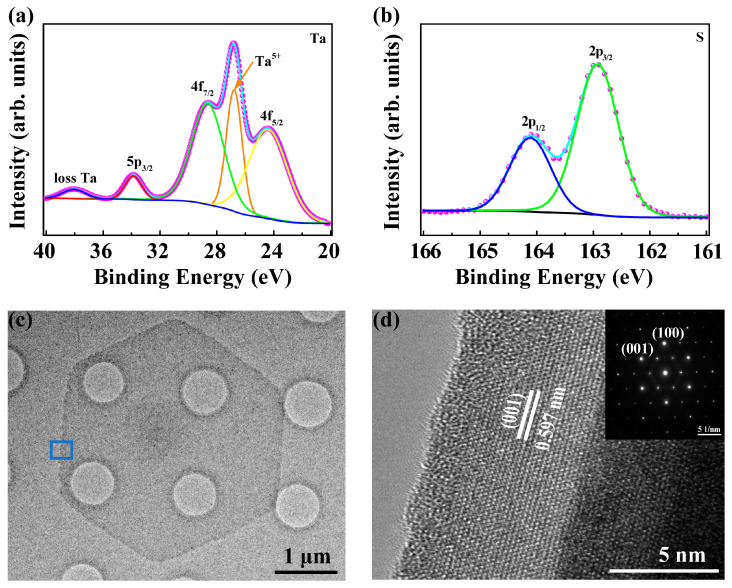
XPS and HRTEM measurements of 1T-TaS_2_ crystal. (**a**,**b**) XPS spectra of Ta-4f, 5p and loss Ta, and S 2p orbitals. (**c**) Low-magnification image of the hexagonal shape sample. (**d**) Atomic-resolution HRTEM image of 1T-TaS_2_ and the inset shows the corresponding SAED pattern.

The controllable synthesis of 1T-TaS_2_ was further studied through AFM measurements, as shown in Figure 2a–d. The line profiles in the inset clearly show the thickness: from the monolayer of 0.7 nm to the tetralayer of 2.9 nm. It should be noted that the sizes of all grown 2D crystals were around 10 μm, which may be limited by the metallic precursor for this APCVD growth. With decreasing thickness, the stability of 1T-TaS_2_ becomes worse. The instability was further confirmed by the XPS measurements in Figure 3a. Besides the Ta-4f_7/2_ peak at 24.2 eV for TaS_2_, peaks of Ta-4f_5/2_ at 26.8 eV, and peaks of Ta-4f_7/2_ at 28.71 eV, a loss feature at about 38 eV for Ta_2_O_5_ also appears in the XPS spectrum, indicating the oxidization of 1T-TaS_2_. The S-2p_1/2_ and S-2p_3/2_ are shown in Figure 3b, giving the evidence for TaS_2_ [39]. For the monolayer, it is even difficult to perform Raman measurements on it due to the low laser-induced damage threshold. Thus, most of the measurements were performed on the thick 1T-TaS_2_ crystals.

HRTEM measurements were performed to explore the internal structure and crystallinity of the sample. The as-grown 1T-TaS_2_ was transferred to a micro-grid-supported carbon film via a PMMA-assisted transfer method [36]. As shown in Figure 3c, the low-magnification HRTEM image depicts the hexagonal shape of the sample with a uniform surface. HRTEM measurements were performed on the area marked in Figure 3c, and the corresponding atomic-resolution image is shown in Figure 3d. The SAED pattern shows the (100) and (001) with the spacing of 0.291 and 0.597 nm in the inset of Figure 3d, agreeing with the lattice spacing of 1T-TaS_2_ [40]. In addition, the single clear dots indicate the high crystalline quality of the grown sample. 

**Figure 4:**
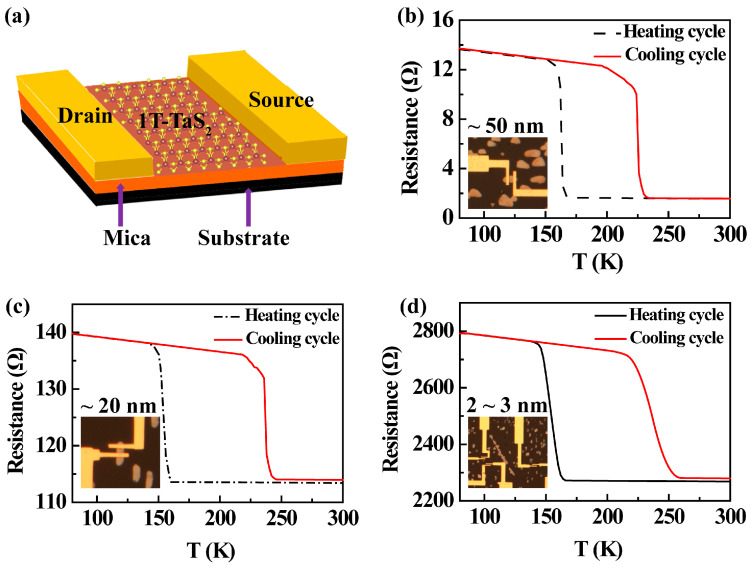
Temperature-dependent resistance measurements. (**a**) Schematic diagram of electrical devices. (**b**–**d**) Temperature-dependent resistance of samples with thicknesses of ~50 nm, ~20 nm, and 2–3 nm during the heating and cooling. The insets are microscopic photographs of electrical devices.

To study the CDW phase transitions, electrical devices were fabricated. In our experiments, it is very difficult to grow large-sized thin 1T-TaS_2_ crystals. Usually, the size of a 1T-TaS_2_ crystal with a thickness lower than 3 nm is below 10 μm. Meanwhile, the thin 1T-TaS_2_ is not so stable in the atmosphere. Hence, it is challenging to fabricate four contacts on a thin sample with a size smaller than 10 μm. To better compare the test results from samples with different thicknesses, two-contact devices were fabricated for all samples, which were proven to be valid for the resistance measurements on exfoliated samples [16]. During the electrical measurements, the positive and negative electrodes were fixed at different temperatures to prevent inconsistent modifications to hysteresis loops from Schottky effects. When the CDW phase transition occurs, the 1T-TaS_2_ phase can change from an insulator to a metal, which can be identified by temperature-dependent resistance measurements. The schematics for the electrical devices are presented in Figure 4a, in which gold electrodes were fabricated on the sample by lithography termina, and more details for the procedure refer to the method. The hysteresis loops of the resistance in the heating and cooling cycles of 1T-TaS_2_ with different thicknesses were measured, as shown in Figure 4b–d. In Figure 4b, the resistance of the sample with a thickness of about 50 nm decreases sharply when the temperature exceeds 160 K during heating. The resistance changing with the temperature indicates the phase transition from the CCDW phase to the NCDW phase.

On the other hand, when the temperature decreases from 300 K to 225 K, the resistance suddenly increases, indicating the occurrence of the NC–CC CDW phase transition. Similar results have been obtained from other samples with different thicknesses. In the sample with a thickness of about 20 nm in Figure 4c, the CC–NC phase transition happens at 153 K, while the NC–CC phase transition occurs at 237 K. Moreover, in the sample with a 2–3 nm thickness shown in Figure 4d, the resistance begins to decrease at 150 K during the heating process for the CC–NC phase transition, while the NC–CC phase transition temperature happens at 250 K.

The above measurements were repeated more than 10 times, and the same conclusions were obtained. It is found that during the heating process, with the increase in thickness, the temperature for the CCDW-NCDW phase transition increased and ranged from 150 K to 160 K. During the cooling process, the NC-CC phase transition temperature decreased with increasing thickness, ranging from 250 K to 220 K. As the thickness decreases, the window for the hysteresis loop becomes larger, which may be attributed to the larger NC–CC phase transition barrier in thinner 1T-TaS_2_ flakes due to the enhanced pinning of nucleated domain walls [10,41]. Such hysteresis loops were suggested to be caused by the domain wall propagation, leading to the ferroic performance of 2D 1T-TaS_2_ crystals [16]. These ferro-rotational orders can be switched by controlling the applied voltages at a fixed temperature or the temperature at a fixed applied voltage. Therefore, thermal-driven resistance switching and the temperature of the thickness-dependent phase transition could extend the electrical and magnetic application of 1T-TaS_2_.

**Figure 5:**
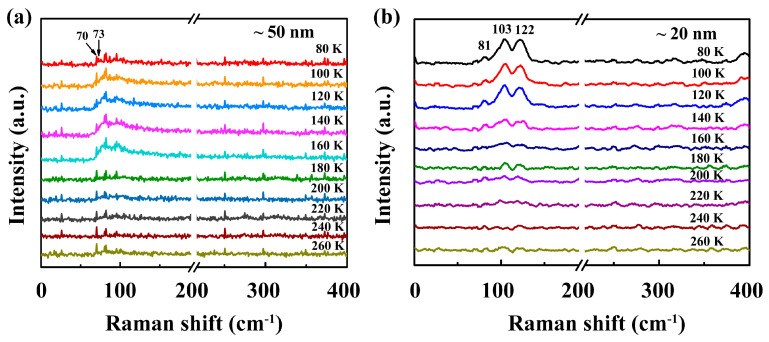
Raman measurements during thermal−driven phase transitions in 1T-TaS_2_. (**a**,**b**) Raman characteristics of CC/NC phase transition on ~50 nm and ~20 nm samples. The Raman spectra were collected at measured temperatures rising from 80 K to 260 K.

The phase transitions were further confirmed by the temperature-dependent Raman measurements. The samples grown on mica were quickly transferred to a vacuum thermostat with a temperature range from 80 K to 260 K to collect the temperature-dependent Raman spectra. Figure 5a shows the Raman spectra of the 50 nm thick sample, indicating an obvious phase transition with emerging new peaks at 70 cm^−1^ and near 100 cm^−1^ as it warmed up. At 160 K, the peak at 70 cm^−1^ was split into two peaks at 70 cm^−1^ and 73 cm^−1^, while the peak intensities were significantly increased near 100 cm^−1^. Those changes are provoked by the folding of phonon modes in the Brillouin zone between the CC/NC translation [42]. Figure 5b shows the Raman spectra of the 20 nm thick sample as it warmed up. It is obvious that the peak intensities were strongly enhanced near 100 cm^−1^ at below 140 K, including the peaks at 81 cm^−1^, 103 cm^−1^, and 122 cm^−1^, but no split peak was seen in the Raman spectra. Furthermore, the same measurements were performed on 2–3 nm thick samples, but no apparent phase transition was found, which may be interpreted as the instability of ultrathin TaS_2_ under the irradiation of lasers. Such vanishment was also reported by previous work in TaS_2_ sheets thinner than 13 nm [43,44]. The above temperature-dependent Raman measurements also confirm that the CC/NC transition temperature in thick layer samples is 140–160 K. The consistency between the resistance measurements and Raman spectra further confirms that the two-contact measurements are reasonable in our experiments.

## 4. Conclusions

High-quality 2D 1T-TaS_2_ crystals were successfully synthesized by CVD with controllable layer numbers. The structural tests show the high quality of the grown samples. The AFM measurements demonstrated the precise control of the thickness. Based on the as-grown samples, their thickness-dependent CC/NCDW phase transitions were revealed by the combination of the temperature-dependent resistance measurements and Raman spectra. The phase transition temperature increases with increasing thickness. The transition hysteresis loops due to temperature-dependent resistance changes of 1T-TaS_2_ can be used for memory devices and oscillators, which is promising for various electrical applications.

## Data Availability

Data available on request due to restrictions e.g., privacy or ethical.

## References

[1] Peng J., Yu Z., Wu J., Zhou Y., Guo Y., Li Z., Zhao J., Wu C., Xie Y. (2018). Disorder enhanced superconductivity toward TaS_2_ monolayer. ACS Nano.

[2] Wen W., Zhu Y., Dang C., Chen W., Xie L. (2019). Raman spectroscopic and dynamic electrical investigation of multi-state charge-wave-density phase transitions in 1T-TaS_2_. Nano Lett..

[3] Nakata Y., Sugawara K., Chainani A., Oka H., Bao C.H., Zhou S.H., Chuang P.Y., Cheng C.M., Kawakami T., Saruta Y. (2021). Robust charge-density wave strengthened by electron correlations in monolayer 1T-TaSe_2_ and 1T-NbSe_2_. Nat. Commun..

[4] Zhao X., Song P., Wang C., Riis-Jensen A.C., Fu W., Deng Y., Wan D., Kang L., Ning S., Dan J. (2020). Engineering covalently bonded 2D layered materials by self-intercalation. Nature.

[5] Wang P., Huan Y., Yang P., Cheng M., Shi J., Zhang Y. (2021). Controlled syntheses and multifunctional applications of two-dimensional metallic transition metal dichalcogenides. Acc. Mater. Res..

[6] Zhu C., Chen Y., Liu F., Zheng S., Li X., Chaturvedi A., Zhou J., Fu Q., He Y., Zeng Q. (2018). Light-tunable 1T-TaS_2_ charge-density-wave oscillators. ACS Nano.

[7] Yoshida M., Suzuki R., Zhang Y., Nakano M., Iwasa Y. (2015). Memristive phase switching in two-dimensional 1T-TaS_2_ crystals. Sci. Adv..

[8] Afzali M., Mostafavi A., Shamspur T. (2020). Improved perovskite solar cell with 2H–TaS_2_ nanosheets as an electron transport layer using microwave irradiation. J. Alloys Compd..

[9] Feng Y., Gong S., Du E., Yu K., Ren J., Wang Z., Zhu Z. (2019). TaS_2_ nanosheet-based ultrafast response and flexible humidity sensor for multifunctional applications. J. Mater. Chem. C.

[10] Huan Y., Shi J., Zou X., Gong Y., Zhang Z., Li M., Zhao L., Xu R., Jiang S., Zhou X. (2018). Vertical 1T-TaS_2_ synthesis on nanoporous gold for high-performance electrocatalytic applications. Adv. Mater..

[11] Ekoya B.G.M., Shan Y.B., Cai Y.C., Okombi N.I., Yue X.F., Xu M.S., Cong C.X., Hu L.G., Qiu Z.J., Liu R. (2022). 2H Tantalum disulfide nanosheets as substrates for ultrasensitive SERS-based sensing. ACS Appl. Nano Mater..

[12] Jarach Y., Rodes L., Ber E., Yalon E., Kanigel A. (2022). Joule-heating induced phase transition in 1T-TaS_2_ near room temperature probed by thermal imaging of power dissipation. Appl. Phys. Lett..

[13] Jia Y., Liao Y.L., Cai H.Z. (2022). High quality TaS_2_ nanosheet SPR biosensors improved sensitivity and the experimental demonstration for the Ddetection of Hg^2+^. Nanomaterials.

[14] Ishiguro Y., Suzuki R., Yangzhou Z., Kodama N., Takai K. (2023). Correlation between charge density wave phase transition and hydrogen adsorption in 1T-TaS_2_ thin film devices. Nanotechnology.

[15] Buravets V., Hosek F., Lapcak L., Miliutina E., Sajdl P., Elashnikov R., Svorcik V., Lyutakov O. (2023). Beyond the platinum era-scalable preparation and electrochemical activation of TaS_2_ flakes. ACS Appl. Mater. Interfaces.

[16] Liu G., Qiu T., He K., Liu Y., Lin D., Ma Z., Huang Z., Tang W., Xu J., Watanabe K. (2023). Electrical switching of ferro-rotational order in nanometre-thick 1T-TaS_2_ crystals. Nat. Nanotechnol..

[17] Sugawara K., Nakata Y., Fujii K., Nakayama K., Souma S., Takahashi T., Sato T. (2019). Monolayer VTe_2_: Incommensurate fermi surface nesting and suppression of charge density waves. Phys. Rev. B.

[18] Lian C.-S., Si C., Duan W. (2018). Unveiling charge-density wave, superconductivity, and their competitive nature in two-dimensional NbSe_2_. Nano Lett..

[19] Sipos B., Kusmartseva A.F., Akrap A., Berger H., Forró L., Tutiš E. (2008). From mott state to superconductivity in 1T-TaS_2_. Nat. Mater..

[20] Vaskivskyi I., Gospodaric J., Brazovskii S., Svetin D., Sutar P., Goreshnik E., Mihailovic I.A., Mertelj T., Mihailovic D. (2015). Controlling the metal-to-insulator relaxation of the metastable hidden quantum state in 1T-TaS_2_. Sci. Adv..

[21] Liu G., Rumyantsev S., Bloodgood M.A., Salguero T.T., Balandin A.A. (2018). Low-frequency current fluctuations and sliding of the charge density waves in two-dimensional materials. Nano Lett..

[22] Gao J., Park J.W., Kim K., Song S.K., Park H.R., Lee J., Park J., Chen F., Luo X., Sun Y. (2020). Pseudogap and weak multifractality in 2D disordered mott charge-density-wave insulator. Nano Lett..

[23] Bu K., Zhang W., Fei Y., Wu Z., Zheng Y., Gao J., Luo X., Sun Y.-P., Yin Y. (2019). Possible strain induced mott gap collapse in 1T-TaS_2_. Commun. Phys..

[24] Perfetti L., Loukakos P., Lisowski M., Bovensiepen U., Berger H., Biermann S., Cornaglia P., Georges A., Wolf M. (2006). Time evolution of the electronic structure of 1T−TaS_2_ through the insulator-metal transition. Phys. Rev. Lett..

[25] Stojchevska L., Vaskivskyi I., Mertelj T., Kusar P., Svetin D., Brazovskii S., Mihailovic D. (2014). Ultrafast switching to a stable hidden quantum state in an electronic crystal. Science.

[26] Vaskivskyi I., Mihailovic I., Brazovskii S., Gospodaric J., Mertelj T., Svetin D., Sutar P., Mihailovic D. (2016). Fast electronic resistance switching involving hidden charge density wave states. Nat. Commun..

[27] Ma L., Ye C., Yu Y., Lu X.F., Niu X., Kim S., Feng D., Tománek D., Son Y.-W., Chen X.H. (2016). A metallic mosaic phase and the origin of mott-insulating state in 1T-TaS_2_. Nat. Commun..

[28] Wu D., Ma Y., Niu Y., Liu Q., Dong T., Zhang S., Niu J., Zhou H., Wei J., Wang Y. (2018). Ultrabroadband photosensitivity from visible to terahertz at room temperature. Sci. Adv..

[29] Shi J., Wang X., Zhang S., Xiao L., Huan Y., Gong Y., Zhang Z., Li Y., Zhou X., Hong M. (2017). Two-dimensional metallic tantalum disulfide as a hydrogen evolution catalyst. Nat. Commun..

[30] Pathan M.A.K., Gupta A., Vaida M.E. (2021). Exploring the growth and oxidation of 2D-TaS_2_ on Cu(111). Nanotechnology.

[31] Dombrowski D., Samad A., Murray C., Petrovic M., Ewen P., Michely T., Kralj M., Schwingenschlogl U., Busse C. (2021). Two phases of monolayer tantalum sulfide on Au(111). ACS Nano.

[32] Fu W., Chen Y., Lin J., Wang X., Zeng Q., Zhou J., Zheng L., Wang H., He Y., He H. (2016). Controlled synthesis of atomically thin 1T-TaS_2_ for tunable charge density wave phase transitions. Chem. Mater..

[33] Wang X., Liu H., Wu J., Lin J., He W., Wang H., Shi X., Suenaga K., Xie L. (2018). Chemical growth of 1T-TaS_2_ monolayer and thin films: Robust charge density wave transitions and high bolometric responsivity. Adv. Mater..

[34] Zhao R., Wang Y., Deng D., Luo X., Lu W.J., Sun Y.-P., Liu Z.-K., Chen L.-Q., Robinson J. (2017). Tuning phase transitions in 1T-TaS_2_ via the substrate. Nano Lett..

[35] Neto A.C., Novoselov K. (2011). New directions in science and technology: Two-dimensional crystals. Rep. Prog. Phys..

[36] Wang K., Huang B., Tian M., Ceballos F., Lin M.-W., Mahjouri-Samani M., Boulesbaa A., Puretzky A.A., Rouleau C.M., Yoon M. (2016). Interlayer coupling in twisted WSe_2_/WS_2_ bilayer heterostructures revealed by optical spectroscopy. ACS Nano.

[37] Duffey J., Kirby R., Coleman R. (1976). Raman scattering from 1T-TaS_2_. Solid State Commun..

[38] Cho D., Cheon S., Kim K.-S., Lee S.-H., Cho Y.-H., Cheong S.-W., Yeom H.W. (2016). Nanoscale manipulation of the mott insulating state coupled to charge order in 1T-TaS_2_. Nat. Commun..

[39] Eda G., Yamaguchi H., Voiry D., Fujita T., Chen M., Chhowalla M. (2011). Photoluminescence from chemically exfoliated MoS_2_. Nano Lett..

[40] Cain J.D., Oh S., Azizi A., Stonemeyer S., Dogan M., Thiel M., Ercius P., Cohen M.L., Zettl A. (2021). Ultranarrow TaS_2_ Nanoribbons. Nano Lett..

[41] He R., Okamoto J., Ye Z., Ye G., Anderson H., Dai X., Wu X., Hu J., Liu Y., Lu W. (2016). Distinct surface and bulk charge density waves in ultrathin 1T−TaS_2_. Phys. Rev. B.

[42] Grisafe B., Zhao R., Ghosh R.K., Robinson J.A., Datta S. (2018). Electrically triggered insulator-to-metal phase transition in two-dimensional (2D) heterostructures. Appl. Phys. Lett..

[43] Yu Y., Yang F., Lu X.F., Yan Y.J., Cho Y.-H., Ma L., Niu X., Kim S., Son Y.-W., Feng D. (2015). Gate-tunable phase transitions in thin flakes of 1T-TaS_2_. Nat. Nanotechnol..

[44] Darancet P., Millis A.J., Marianetti C.A. (2014). Three-dimensional metallic and two-dimensional insulating behavior in octahedral tantalum dichalcogenides. Phys. Rev. B.

